# High Dynamic Micro Vibrator with Integrated Optical Displacement Detector for In-Situ Self-Calibration of MEMS Inertial Sensors

**DOI:** 10.3390/s18072055

**Published:** 2018-06-27

**Authors:** Yi-Jia Du, Ting-Ting Yang, Dong-Dong Gong, Yi-Cheng Wang, Xiang-Yu Sun, Feng Qin, Gang Dai

**Affiliations:** Microsystem and Terahertz Research Center, Institute of Electronic Engineering, China Academy of Engineering Physics, Mianyang 621999, China; duyijia@mtrc.ac.cn (Y.-J.D.); yangtingting@mtrc.ac.cn (T.-T.Y.); gongdongdong@mtrc.ac.cn (D.-D.G.); wangyicheng@mtrc.ac.cn (Y.-C.W.); sunxiangyu@mtrc.ac.cn (X.-Y.S.); qinfeng@mtrc.ac.cn (F.Q.)

**Keywords:** in-situ self-calibration, 6-DOF, micro vibrator, integrated optical detector, high-dynamic

## Abstract

The scale factor drifts and other long-term instability drifts of Micro-Electro-Mechanical System (MEMS) inertial sensors are the main contributors of the position and orientation errors in high dynamic environments. In this paper, a novel high dynamic micro vibrator, which could provide high acceleration and high angular rate rotation with integrated optical displacement detector, is proposed. Commercial MEMS inertial sensors, including 3-axis accelerometer and 6-axis inertial measurement unit which is about 3 mm * 3 mm * 1 mm with 19 mg, could be bonded on the vibration platform of the micro vibrator to perform in-situ during the self-calibration procedure. The high dynamic micro vibrator is fabricated by a fully-integrated MEMS process, including lead zirconate titanate (PZT) film deposition, PZT and electrodes patterning, and structural ion etching. The optical displacement detector, using vertical-cavity surface-emitting laser (VCSEL) and photoelectric diodes (PD), is integrated on the top of the package to measure the 6-DOF vibrating displacement with the detecting resolution of 150 nm in the range of 500 μm. The maximum out-of-plane acceleration of the z-axis vibrating platform loaded with commercial 3-axis accelerometer (H3LIS331DL) achieves above 16 g and the maximum angular velocity achieves above 720°/s when the driving voltage is ±6 V.

## 1. Introduction

The MEMS-based micro-inertial measurement unit (MEMS-IMU) can provide the multi degree-of-freedom (DOF) inertial information required for inertial positioning. With the advantages of small size, light weight, low cost, and easy integration, MEMS-IMU has become the core unit of the micro inertial navigation and positioning systems [1,2]. Although the operational accuracy of micro inertial sensors is approaching the level of macro inertial sensors, the long-term drifts of zero and scale factor still limit their potential in high accuracy strategic and navigation applications, and the calibration validity duration based on traditional calibration methods is quite short [3]. 

With the development of an MEMS process, the methodology, which applies the micro-structures embedded inside or integrated outside to self-calibrate the micro-sensor before working, to compensate the long-term drift error, may fundamentally reduce the dependence on long-term stability, greatly expanding the application of MEMS inertial sensors [4,5,6,7]. Among the recently reported self-test and self-calibration methods for improvement of the long-term drifts of zero and scale factor in MEMS inertial sensors, the micro vibrator capable of providing 6-DOF motion and measuring scale factor nonlinearity, cross-axis coupling error, acceleration, and sensitivity of the gyro, etc., has become a promising solution. 

In 2013, Najafi, et al. applied the 3-DOF micro vibrator to the self-calibration problem of MEMS-IMU and microgyroscopes for the first time [8]. The main idea is to drive the vibrator to provide physical excitation for the MEMS-IMU, and simultaneously detect the trajectory of the MEMS-IMU to calibrate it. The platform consists of a 3-DOF micromotion stage that can provide piezoelectric actuation for Z-translation and X/Y-tilting reference stimuli with a small off-axis motion compared to the preferred direction of actuation. The following research works are mainly focused on the in-situ calibration method for the gyroscope as well as the fabrication of larger micro vibrators [9,10,11,12,13,14]. Further, the transient linear acceleration and angular velocity output of those vibrators grow continuously up to 0.3 g [15] and 280°/s [10], respectively. For applications requiring dynamic motion for MEMS inertial sensors with high measure range, which need further information including scale factor at high g and rotation rate, nonlinearity and so on, higher linear acceleration and angular velocity output are expected.

To obtain the exact position of the micro-vibrator and the MEMS-IMU payload is critical for in-situ self-calibration. Najafi, et al. proposed a piezoelectric differential sensing method in which the crab claw beams of the vibrator can be selected as either a driving or detection unit [10]. The system architecture is simple without the need for an additional sensor section, but the detection accuracy and repeatability are not high. Another method of motion detection for MEMS-IMU is based on capacitive sensing, which provides a threshold point with very high precision in the case of skillfully imbedding the capacitor plate electrodes at the bottom side of the vibratory stage and substrate [11,14]. When the detected current changes from positive to negative, the corresponding displacement is considered to be accurate and a threshold signal is given. However, the design of the capacitive threshold sensor needs to be closely combined with the design of the micro-vibrator, which increases the complexity of the design and implementation of the vibrator and is not conducive to ensuring the accuracy and reliability of the calibration. Micro-optical displacement sensors based on MEMS technology can realize higher-precision motion detection compared to the capacitive counterpart [16,17,18]. Integrated micro-displacement sensor to measure 2-DOF micro-motion has been reported [18], but there is currently no motion detection report based on a miniature optical displacement sensor dedicated to measure the acceleration and angular velocity of micro-vibrator.

In this paper, a new, easy-to-integrate, high-dynamic, microvibratory actuation and optical sensing platform for in-situ self-calibration of the commercial MEMS inertial sensors is presented. This high-dynamic micro-vibrator was fabricated by a fully integrated MEMS process, including PZT film deposition, PZT and electrodes patterning, and structural ion etching. Within our limited knowledge, this is the first time to report a micro-vibrator with integrated optical displacement detection. The micro-vibrator has been designed to provide 6-DOF piezoelectric actuation excitation with measured maximum z-axis out-of-plane acceleration of above 16 g and the maximum X/Y angular velocity of above 720°/s when a 19 mg commercial MEMS inertial sensor is mounted. Such a high linear acceleration and angular velocity provided by the micro-vibrator is beneficial to satisfy the requirement of the MEMS-IMU targeting high-g applications. The optical displacement detector consists of a VCSEL and eight PDs, and is bonded and connected on the silicon substrate to form a monolithic optical beam deflection method (OBDM) chip. The OBDM chip is not in contact with the micro vibrator physically, which greatly reduces the process complexity and assembly difficulty while maintaining the high precision for motion measurement (resolution of 150 nm in the range of 500 μm). This proposed platform can potentially be adapted as a universal system-in-package solution to provide precise on-chip physical reference inputs and in-situ self-calibration of scale factor drifts for multi-types and multi-structures of MEMS inertial sensors including accelerometers, gyroscopes, and other IMUs.

## 2. Microsystem Architecture for In-Situ Self-Calibration of MEMS Inertial Sensors

**Figure 1:**
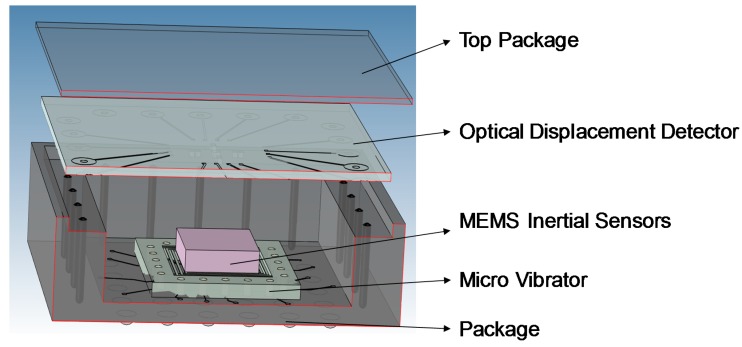
Graphical overview of the proposed microsystem architecture for in-situ self-calibration of MEMS inertial sensors.

The schematic diagram of the microsystem architecture is illustrated in Figure 1. The micro vibrator is integrated with the MEMS-IMU through system-in-package (SiP) technology, and produce the required reference excitation signals while not causing any degradation in MEMS-IMU performance. The monolithic OBDM detector is located on the cover plate of the package, spatially on top of MEMS inertial sensors to provide precise motion sensing and estimation of the target inertial sensor. 

Firstly, the vibrator is driven to provide a high-g or high angular velocity vibration for the MEMS-IMU. The matchup relations between the driving voltage for PZT and the linear reference acceleration or angular velocity is built before self-calibration. And the input reference acceleration or angular velocity provided by the micro vibrator could be adjusted by controlling the amplitude and the frequency of the driving voltage. Then, the MEMS-IMU motion trajectory is precisely monitored by the OBMD detector with a closed-loop control algorithm. By comparing the output results of the MEMS sensor with reference acceleration and angular velocity, the calibration and compensation for the long-term drift error output of the target inertial sensor is carried out, achieving in-situ self-calibration of MEMS inertial sensors finally.

## 3. 6-DOF High Dynamic Micro-Vibrator for On-Chip Physical Stimulus

Increasing the angular velocity and linear acceleration range that micro-vibrator can provide is the key to verify the performance and effectiveness of MEMS inertial sensors at high g and high angular velocities. Linear acceleration range also determines the accuracy of identifying error parameters of the self-calibration system, helping to obtain long-term drift information of sensor scale factor. Therefore, it is the goal of this paper to obtain high acceleration and angular velocity for micro-vibrator under loading conditions and low voltage driving.

**Figure 2:**
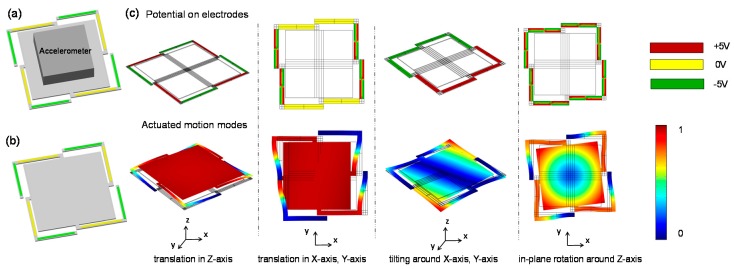
Piezoelectric micro-vibrator with simulated 6-DOF motion capability. (**a**) A schematic of the vibratory platform with an accelerometer payload; (**b**) A finite element method simulation model; (**c**) FEM simulation results for micro-vibrator when the stage is excited for 6-DOF motion.

The acceleration of vibrator is proportional to the displacement amplitude and the square of its characteristic frequency [19]. The angular velocity of the vibrator increases as the characteristic frequency and the maximum angle increase. Thus, increasing the resonant frequencies, the maximum amplitude of the displacement and the maximum angle to achieve higher dynamic range become crucial. Taking the Z-axis linear acceleration optimization as an example, there is a common trade-off in the improvement of resonant frequency and displacement amplitude. For instance, as the device stiffness increases, resonant frequency increases while the displacement amplitude decreases. Since the acceleration is proportional to the square of the frequency, priority is given to the frequency optimization. Therefore, the MEMS micro-vibrator, consisting of a center vibration stage and four L-shaped suspension beams with an accelerometer payload as a mass block connected rigidly on the top surface of the center stage, is presented as shown in Figure 2a. Each suspension beam consists of two piezoelectric unimorph actuator units, which are actuated by application of a vertical electric field across the piezoelectric layer. The connection point between the center stage and the suspension beam is located in the middle of each side of the center stage.

A finite element method simulation model without payload under ±20 V excitation input is proposed as shown in Figure 2b. The first resonant frequency/Z-axis static displacement of our L-shaped beam structure is changed to 2017 HZ/6.76 μm compared to the crab-leg suspension counterpart of 879 HZ/29.6 μm [8], meeting the requirements of acceleration range improvement. In order to achieve the extended motion capability in multiple axis, the top electrode of each piezoelectric unimorph is divided into four sections which could be applied with independent excitation input. 6-DOF-motion modes could be obtained by applying different excitation polarities on these electrodes and their simulated 6-DOF actuation of the vibration platform by distributed-electrode excitation are shown in Figure 2c. These 6-DOF-motion modes are translation in X-axis, Y-axis and Z-axis, and tilting around X-axis, Y-axis as well as in-plane rotation around Z-axis, respectively. Such multi-axis driving capability enables not only on-chip scale factor and offset values calibration of single-axis sensors, but also multi-axis sensors as well as the cross-axis sensitivity of inertial sensors.

**Figure 3:**
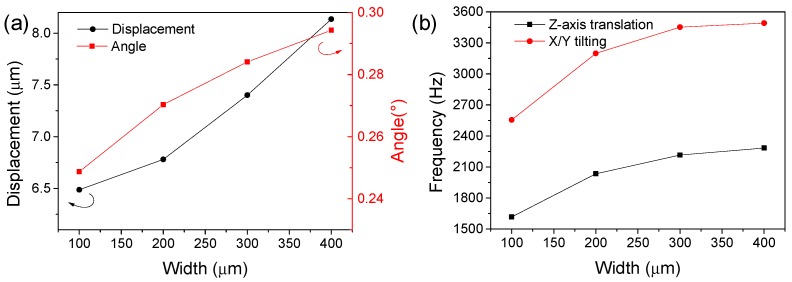
The effect of the width of beam on the (**a**) z-axis translation modal displacement, X/Y tilting inclination angle and (**b**) their corresponding resonant frequency.

The performance of the vibrator could be further improved by adjusting the PZT thickness, size length of the center stage, beam width, etc. Among those variables, increasing the width of beams is an efficient method which causes both resonant frequency and maximum displacement amplitude improvement, since broadened beam results in changes in system stiffness and elasticity coefficient. Figure 3 shows the effect of the width of beam on the Z-axis translation modal displacement, X/Y tilting inclination angle and their corresponding resonant frequency. As can be seen, increasing the width of beam improves greatly the corresponding out-of-plane displacement and inclination angle because the wider beam corresponds to a wider electrode area with stronger driving capability. In terms of frequency characteristics, the beam width also brings about an increase in the modal resonant frequency, which is very favorable for enlarging the working bandwidth of the micro-vibrator.

The MEMS micro-vibrator was fabricated by fully integrated MEMS process, including PZT film deposition, PZT and electrodes patterning and structural ion etching with center stage side length of 3.2 mm, beam width of 200 μm, beam gap of 50 μm, PZT thickness of 5 μm and silicon layer thickness of 25 μm. In addition, the accelerometer's signal wire was connected to the substrate via the L-shaped beams. After the deposition of silicon dioxide for isolation, the accelerometer signal lines were connected to the center stage. And the wiring of the top excitation electrodes and other interconnect electrodes were completed by double layer wiring process. 

## 4. Integrated Optical Displacement Detector

When performing in-situ diagnosis and self-calibration procedure, it is critical to accurately identify the position of the vibration stage of the micro-vibrator which is used as the motion reference and closed-loop control input. There are two common methods to achieve the high-resolution displacement detection, namely capacitive sensing and optical sensing. The capacitive sensing method could provide a threshold point with very high precision and achieve nanometer precision displacement detection. However, the multi-capacitor integration in the micro-vibration stage greatly increases the process difficulty of the vibrator itself. Meanwhile, the capacitor area is so small that the capacitance value is only 0.05 pf level, which greatly increases the difficulty of signal noise processing. Besides, the capacitance method for threshold detection is not suitable for Z-axis translational motion detection. 

The optical-based displacement measurement method has a nanometer displacement detection accuracy. Moreover, the optical detector and the detected object are physically independent, which is beneficial to improve the package consistency and product yield without needs for changing the original process of both. The output signal is moderate and can be processed by a typical PD low noise amplification processing circuit. Besides, it can be used for translational displacement and angular rotation detection.

**Figure 4:**
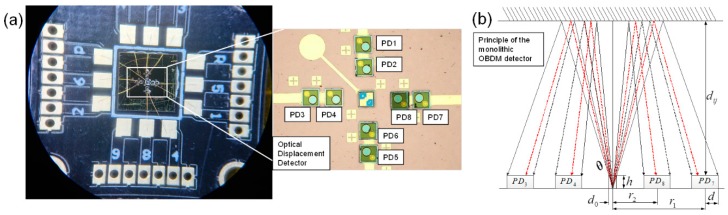
OBDM detector and its working principle. (**a**) The layout of the PDs and VCSEL; (**b**) Schematic of the principle of the monolithic OBDM detector.

Therefore, this paper proposes a high resolution OBDM detector consisting of one VCSEL and eight PDs for micro-vibrator motion measurement, as shown in Figure 4a. The micro vibrator, the target sensor and the OBDM detector are 3D integrated via SiP technology. The OBDM detector is located above the target inertial sensor at a suitable distance and a light reflector is placed on the top surface of the target sensor. The relative motion between the reflector and OBMD detector affect the intensity distribution of the reflected light that could be extracted by different PDs to obtain the changes in micro-displacement and micro-angle simultaneously.

Figure 4b shows the OBDM detector with the layout of PD-arrays and VCSEL. The effective radius of VCSEL and PDs are marked as d0 and d, respectively. The divergence angle of the light is marked as *θ*. And the radius of internal and external PDs are labeled as *r*_1_ and *r*_2_, respectively. The light emitted by the VCSEL reaches the top surface of the target inertial sensor and becomes reflected, and the reflected light completely covers the photosensitive region of the PDs, the received light intensity for PD can be expressed as follows [20]:

(1) $$\;I=\frac{2P_{0}S}{\pi R^{2}}\exp\left[\frac{-2r_{1}{}^{2}}{R^{2}}\right],$$

where R = (2d*_ij_* + z_0_) × tan*θ*, tan*θ* = d_0_/z_0_, S = *π*d_2_, *P*_0_ is the beam power of VCSEL and I is the beam receiving power of PD. According to the above equation, the beam receiving power of the 8 PDs will change as the relative position or angle of the reflector and the VCSEL varies. Therefore, by measuring the electrical output of 8 PDs, the tilting angles and the z-axis displacement of the vibrator and the target inertial sensors can be obtained. The corresponding angular velocity and linear acceleration can be derived after further calculation. 

The z-axis displacement is related with the sum of the inner ring PD voltages or the outer PD voltages.

(2) $$S=P_{PD1}+P_{PD3}+P_{PD5}+P_{PD7},$$

(3) $$\;S=P_{PD2}+P_{PD4}+P_{PD6}+P_{PD8},$$

The tilting angles are related with the sum-difference ratio (Quotient) of two PD voltages in each direction to achieve high detection resolution.

(4) $$Quotient_{X-axis}=\frac{P_{PD2}-P_{PD6}}{P_{PD2}+P_{PD6}},$$

(5) $$Quotient_{X-axis}=\frac{P_{PD4}-P_{PD8}}{P_{PD4}+P_{PD8}},$$

More details about the layout formula, working principle and fabrication process are referred to our previous work [20].

## 5. Experimental Results and Analysis

### 5.1. Experimental Setup

**Figure 5:**
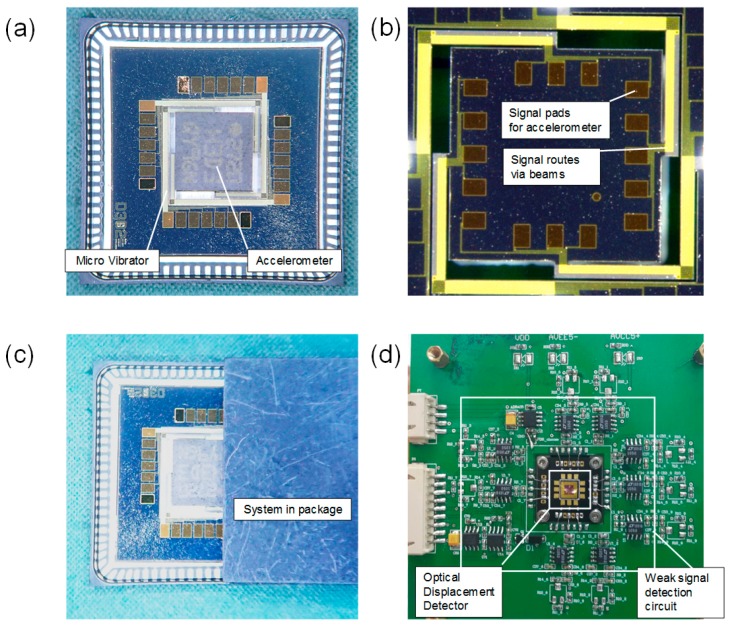
The photos of (**a**) the micro-vibrator with an accelerometer payload, and a light reflector is on the top surface of the accelerometer; (**b**) the micro-vibrator without the accelerometer; (**c**) a system-in-package solution for self-calibration; (**d**) an optical detector with its peripheral signal processing circuit.

In order to verify the dynamic characteristics of the micro vibrator, commercial MEMS 3-axis accelerometer H3LIS331DL, which was about 3 mm * 3 mm * 1 mm with 19 mg, was mounted on the vibration platform, as shown as in Figure 5a. The accelerometer's signal wire was connected to the substrate via the L-shaped beams as shown in Figure 5b. The piezoelectric ac actuation signals were provided individually for each top excitation electrode with different excitation input modes to provide desired multi-DOF motion. The corresponding vibration frequency and amplitude responses of the micro-vibratory stage were characterized via a laser doppler vibrometer which provides a reference standard for the following integrated optical sensor to detect the vibrator motion. The optical sensor was located on the cover plate of the package and then integrated with the vibrator to form a system-in-package solution for self-calibration, as shown in Figure 5c. Finally, the optical detector output was collected and processed by a peripheral circuit and a signal processing system as shown in Figure 5d. 

### 5.2. Actuation and Sensing Performance

**Figure 6:**
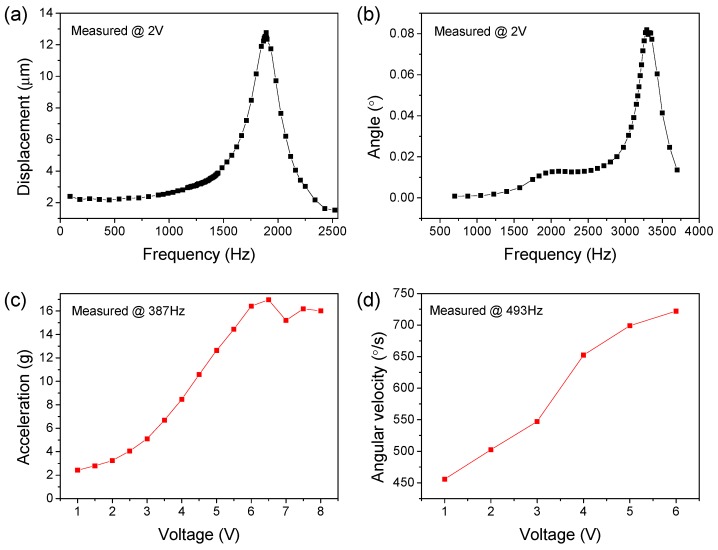
Measured dynamic response of the micro-vibrator via laser doppler vibrometer. (**a**) The amplitude-frequency characteristics and (**b**) angle-frequency characteristics of the unloaded vibrator. (**c**) The Z-axis non-resonant linear acceleration and (**d**) the non-resonant X/Y tilting angular velocity of the loaded vibratory stage is dependent on the excitation voltage.

Firstly, the dynamic response of the vibratory stage without a commercial accelerometer load was characterized via LDVs. The accurate evaluation of dynamic performance could serve as a reference for later optical detection, and also help verify the consistency of simulation design and physical testing. The amplitude-frequency characteristics of the unloaded vibrator is shown in Figure 6a, when sine excitation voltages within ±6 V at different frequency were applied to trigger the Z-axis out of plane motion mode. The vibrator provided a first-order resonant frequency of 1890 Hz which is very close to the simulation result of 2017 Hz, and a maximum Z-axis displacement amplitude measured at resonance point of 12.8 μm. Before resonance, a relatively flat rise trend of the amplitude-frequency curve was observed, which was beneficial to expand the dynamic operating bandwidth of the micro-vibrator. The XY plane tilting motion of vibrator was also tested with ±6 V ac excitation, showing a tilting mode resonance at 3290 Hz with a maximum inclination angle of 0.082° as shown in Figure 6b. The corresponding simulation result shows a close resonant frequency of 3178 Hz. In addition, the Z-axis displacement amplitude or the X/Y tilting angle has a lot of room for further growth as the ac excitation voltage increases.

Compared with the no-load condition, the dynamic response of the vibratory stage after loading can better reflect its calibration ability to the IMU. After finely welding the accelerometer (19 mg) on the metal pad in the center of the stage, the vibration characteristics of the vibrator were measured. The non-resonant linear acceleration in the z-axis translation displacement mode could reach up to 16 g, and increases linearly with the increase of the excitation voltage, as shown in Figure 6c. The non-resonant angular velocity reached up to 720°/s in the X/Y tilting mode, and it also showed a good linear relationship with the excitation voltage, as shown in Figure 6d. In addition, excessive excitation voltage leads to distortion of the waveform and structural nonlinearity problems arise. Therefore, the maximum permissible excitation voltage is determined.

**Figure 7:**
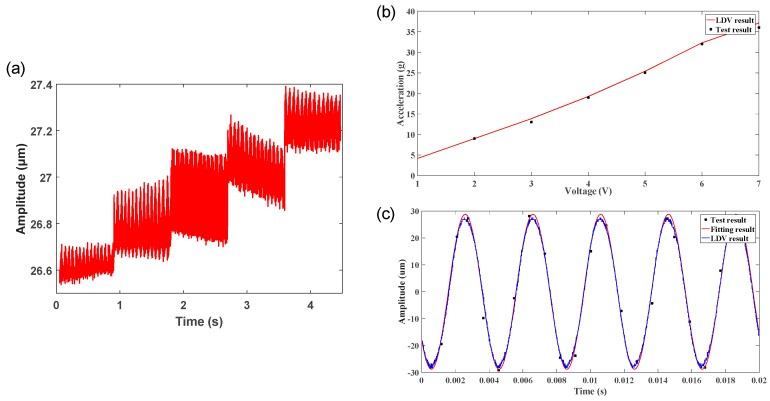
The measurement results comparison of LDVs versus OBDM detector. (**a**) Resolution measurement with steps of 150 nm. (**b**) Calculated acceleration output for increasing periodic excitations on the stage. (**c**) Measured amplitude output with time.

These test results provide a reference standard for the following integrated optical sensor to detect the vibrator motion. A custom-made optical displacement detector was applied to access the exact position of the vibratory stage of the micro vibrator which was used as the motion reference and the input of closed loop control. To evaluate the performance of the optical detector, the vibrator with a commercial accelerometer payload (19 mg) was used as the vibration source and LDVs was used for error assessment of the optical detector. As previously reported [20], a quasi-static test indicates the measurement range can reach 500 μm for the Z-axis translation displacement mode. For demonstration of limit of resolution measurements, dynamic vibration with amplitude steps of 150 nm were generated by the vibrator and detected by the optical detector with the raw measurements plotted in Figure 7a which shows a good differentiation between each step. For demonstration of in situ optical measurement using the OBDM detector, the stage was excited at stepwise incremented amplitudes at a constant frequency (250 Hz), while the OBDM output signal was recorded simultaneously. The calculated acceleration monotonously increases for each excitation step as shown in Figure 7b. For demonstration of the error assessment of the optical detector, the dynamic vibration displacement changes (frequency of 250 Hz) with time measured by LDVs versus the fitting data processed by a state-estimation algorithm from the optical detector output is shown in Figure 7c, which reveals a good match between both. The current measurement resolution of the optical detector could be further improved through the testing system optimization and low-noise circuit optimization, such as a smoothing filtering algorithm or LNA (low noise amplifier). Another important factor determining the measurement resolution is that the light reflected by the reflector will cause oscillations for the VCSEL, which may be solved in the future by adjusting the optical path or growing certain light-absorbing materials on the reflector surface.

## 6. Conclusions

It is highlighted that this paper integrates the micro vibrator with optical displacement detector for the first time to demonstrate the feasibility of prognostic both high-g and angular rate characteristics of generic MEMS inertial sensors. By structural design and optimization, a simulated 6-DOF micro vibrator, consisting of a center vibration stage and four L-shaped suspension beams, could provide tested both high acceleration (16 g) and high angular rate rotation (720°/s) under 19 mg payload. When compared with previously-reported multi-axis motion stages, the introduced micro-vibrator can provide higher-DOF capability, larger Z-axis linear acceleration, larger X/Y tilting angular velocity in a compact device volume, while requiring smaller actuation voltages. An optical beam deflection method (OBDM) using a photoelectric detector array and VCSEL for micro-vibrator motion measurement was studied, showing a detecting resolution of 150 nm in the range of 500 μm. Future work will focus on the testing of the 6-DOF motion capability of the vibrator, exploration of the six-axis optical sensor design, as well as the closed-loop control algorithms to achieve in-situ self-calibration of MEMS inertial sensors.
